# Origin of structural difference in metabolic networks with respect to temperature

**DOI:** 10.1186/1752-0509-2-82

**Published:** 2008-09-22

**Authors:** Kazuhiro Takemoto, Tatsuya Akutsu

**Affiliations:** 1 Department of Computational Biology, Graduate School of Frontier Sciences, University of Tokyo, Kashiwanoha 5-1-5, Kashiwa, Chiba 277-8561, Japan; 2 Bioinformatics Center, Institute for Chemical Research, Kyoto University, Gokasho, Uji, Kyoto 611-0011, Japan

## Abstract

**Background:**

Metabolism is believed to adaptively shape-shift with changing environment. In recent years, a structural difference with respect to temperature, which is an environmental factor, has been revealed in metabolic networks, implying that metabolic networks transit with temperature. Subsequently, elucidatation of the origin of these structural differences due to temperature is important for understanding the evolution of life. However, the origin has yet to be clarified due to the complexity of metabolic networks.

**Results:**

Consequently, we propose a simple model with a few parameters to explain the transitions. We first present mathematical solutions of this model using mean-field approximation, and demonstrate that this model can reproduce structural properties, such as heterogeneous connectivity and hierarchical modularity, in real metabolic networks both qualitatively and quantitatively. We next show that the model parameters correlate with optimal growth temperature. In addition, we present a relationship between multiple cyclic properties and optimal growth temperature in metabolic networks.

**Conclusion:**

From the proposed model, we find that such structural properties are determined by the emergence of a short-cut path, which reduces the minimum distance between two nodes on a graph. Furthermore, we investigate correlations between model parameters and growth temperature; as a result, we find that the emergence of the short-cut path tends to be inhibited with increasing temperature. In addition, we also find that the short-cut path bypasses a relatively long path at high temperature when the emergence of the new path is not inhibited. Even further, additional network analysis provides convincing evidence of the reliability of the proposed model and its conclusions on the possible origins of differences in metabolic network structure.

## Background

Elucidation of basic design principles behind biological systems is a central topic in the post-genomic era. In particular, it is important to understand the cell's adaptation to environmental changes in not only evolutionary biology but also biotechnology. It is believed that most positively selected mutations cause changes in metabolism, resulting in a better-adapted phenotype from natural history, phylogenetics, genetics, and so on. This is an adaptive evolution. Adaptation to temperature is often discussed when considering the evolution of life, because molecular phylogenetic analyses [1-3] support that organisms living at high temperatures are primeval forms of life. Moreover, heat-loving organisms have a great deal of potential in industry. They provide product materials with poise because they are very stable at normal temperatures. In addition, heat-loving organisms are cost-effective because we can utilize them repetitively due to their stability. Thus, elucidation of differences with respect to temperature and their origin is a major topic in several areas of biology.

Living organisms optimally grow in environments of different temperatures. For example, humans optimally grow in a particular temperature, and cannot grow at very high temperatures. However, heat-loving organisms such as *Methanopyrus kandleri *and *Thermoanaerobacter tengcongensis *optimally grow at high temperatures. In general, living organisms are classified into four classes [4]: Hyperthermophiles (extreme heat-loving), Thermophiles (heat-loving), Mesophiles (grow at moderate temperatures) and Psychrophiles (cold-loving).

Up until now, several works have revealed adaptive differences, as a result of temperature, for structural and sequence properties of transcriptomes and proteomes [5]. For example, guanine-cytosine content correlates with growth temperature in ribonucleic acids (RNAs), and charged residues tend to exist in proteins of thermophiles. In particular, such differences at the transcriptome and proteome level might influence metabolism because proteins play roles of many different enzymes in metabolic reactions. Therefore, we can expect a structural difference in metabolic networks with respect to temperature.

The structure of the metabolic networks for many organisms has recently been investigated. We can obtain a large amount of data on metabolic pathways in many organisms from several databases such as KEGG: Kyoto Encyclopedia of Genes and Genomes [6]. For large-scale networks such as metabolic networks, the structural features were analyzed using statistical mechanics and graph-theoretical techniques [7,8]. In particular, several striking structural properties have recently been found such as heterogeneous connectivity [9], small-worlds [10,11], and hierarchical modularity [12]. These properties are absent in random networks [13].

The heterogeneous connectivity is in the degree distribution, defined as the frequency of nodes with *k *edges, which follows the power law *P *(*k*) ∝ *k*^-*γ*^, where *γ *is a constant, and is empirically found to vary from network to network [7,8,14,15]. This power-law distribution indicates that a few nodes (hubs) integrate a great number of nodes and most of the remaining nodes do not. In addition, the exponent *γ *which is the so-called "degree exponent", reflects a macroscopic tendency of the connectivity in networks. In the case of a large degree exponent, the probability that a node with large degree exists in a network becomes low. That is, most nodes have similar degrees in the networks, indicating that the connectivity of the network is homogeneous. In the case of a small degree exponent, in contrast, nodes tend to have different degrees in the networks, suggesting that the connectivity of the network is heterogeneous, and therefore it is statistically possible to find highly connected nodes or hubs.

The small-world property is reflected in high clustering coefficients *C *[10], which denote the density of edges between neighbors of a given node, and implies the modularity of networks [16]. The modular structures are actively investigated with statistical approaches, and it is found that the degree-dependent clustering coefficient, defined as a correlation between the number of edges *k *of a given node and the clustering coefficient *C *of the node, follows the power law with exponent -1; thus *C*(*k*) ∝ *k*^-1^. The power-law function suggests modules themselves also form a hierarchical structure [12,17].

In recent years, the relationship between such structural properties and optimal growth temperature in metabolic networks has been investigated, and as a result, the structural difference with respect to temperature has been revealed [18]. With increasing tempoerature, the edge density (the ratio of the number of edges to the number of nodes) and the clustering coefficient decrease, and the degree exponent increases. This result implies that metabolic networks transit from heterogeneous and highly clustered (highly modular) structures to homogeneous and less clustered (low modular) structures with increasing temperature. Moreover, the authors have speculated that this structural transition is due to the difference in selective constraints between thermophiles and non-thermophiles [19,20]. However, an assuredness of this hypothesis still not has been shown because of the unclear relationship between the differences in selective constraints and that of resulting structural properties. That is, it is unclear how the difference in selective constrains affects local evolutionary events and consequently influences global network structure. In order to show a more concrete hypothesis, we need to clarify what mechanisms (local rules) determine such structural properties, and need to reveal the relationship between the mechanisms and growth temperature. Consequently, we propose a network model which reproduces the structural properties such as the degree distribution and the degree-dependent clustering coefficient of metabolic networks. Network models are useful to reveal the relationship between local events (microscopic rules) and global (macroscopic) features (structural properties) [7,15]. We try to discuss the origin of structural differences with respect to temperature via the proposed model.

In this paper, we first explain the details of the proposed model with two parameters. We provide mathematical solutions of the model, and explain how to estimate the parameters from real data (see Method for details). Moreover, in order to confirm that the model reproduces structural properties, we compare the model with the real metabolic networks of 113 organisms that were investigated in reference [18]. We next investigate the correlation between the parameters and growth temperature, and present a more concrete hypothesis for the origin of structural differences in metabolic networks with respect to temperature. In addition, we investigate a relationship between cyclic properties and temperature in metabolic networks in order to show more convincing evidence of this hypothesis.

## Results

### Network model

Here, we propose a simple model, which reproduces structural properties of metabolic networks, with two parameters *p *and *q*.

In general, metabolic networks are believed to evolve via gene duplications [21-23] and horizontal gene transfer [24]. Gene duplication is a process in which multiple copies of a DNA fragment emerge in a genome due to mistakes such as DNA replication errors. Horizontal gene transfer is any process in which an organism transfers genetic material to another cell. As a result, these processes often provide new proteins. For this reason, gene duplication and horizontal gene transfer are believed to play major roles in evolution [25,26]. Due to these processes, new reactions often emerge in metabolic networks because proteins play the roles of many types of enzymes. Therefore, metabolic networks are believed to grow via gene duplication and horizontal gene transfer. In this case, we can consider two situations: the case that a new metabolite develops and a corresponding new reaction occurs between it and an existing metabolite (Event I), and the case that a new reaction occurs between existing metabolites (Event II). Here, we assume that a network has a connected component. That is, we do not consider situations that an isolated node connects to an isolated cluster. This is because structural differences are observed in the largest connected components in real metabolic networks. We neglect such situations according to this experimental condition.

Moreover, duplicated proteins might be functionally similar to an original protein. That is, a duplicated enzyme (protein) might catalyze a reaction which is similar to a reaction catalyzed by an original enzyme (protein). Therefore, it is believed that duplicated pairs of enzymes are close to each other in metabolic networks [21,23].

In consideration of the above, we construct a model as follows.

**Figure 1:**
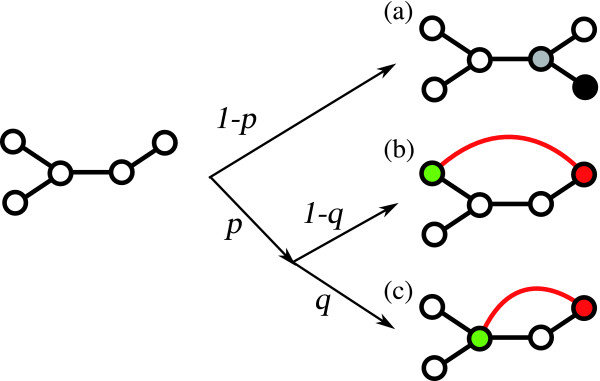
**Schematic diagram of the growth mechanisms of the model**. (a) Event I. The black and gray nodes are a new node and a randomly selected existing node, respectively. (b) and (c) Event II. The red lines correspond to new edges. The red nodes are randomly selected existing nodes. The green nodes are existing nodes, which are selected by a random walk from each red node. The new edge becomes a short-cut path between the red node and the green node.

(i) With probability 1 - *p*, Event I occurs [see Figure 1(a)]. In this case, a new node [the black node in Figure 1(a)] is connected to a randomly selected existing node [the gray node in Figure 1(a)].

(ii) With probability *p*, Event II occurs [see Figures 1(b) and 1(c)]. In this case, a short-cut path bypasses a path between a node and another node. We need to consider the length of the path bypassed. However, when we investigate the degree distribution and the degree-dependent clustering coefficient, it is sufficient to consider only two cases: (1) the case of length 2 and (2) the case that the length is greater than 2. This assumption (of considering only two cases) is appropriate because the degree distribution is independent of the bypassed path length and the clustering coefficient is only influenced in the case that a path of length 2 is bypassed (see the section "Mathematical solution" in Method for details). Therefore, we express the bypassed path length using the parameter *q *as follows.

First, an initial node [the red nodes in Figures 1(b) and 1(c)] is selected at random.

(1) With probability *q*, next, we select a path of length 2 to bypass based on a random walk from the initial node.

(2) With probability 1 - *q*, in contrast, we select a path to bypass whose length is greater than 2 based on a random walk from the initial node.

Thus, the parameter *q *roughly reflects the degree of the bypassed path length. The random walk is considered in order to model the feature that duplicated pairs of enzymes are close to each other as explained above. Finally, a new edge (short-cut path) is drawn between the initial node [the red nodes in Figures 1(b) and 1(c)] and the terminal node [the green nodes in Figures 1(b) and 1(c)]. Note that a triangle is accordingly generated with the probability *p *× *q*.

Using mean-field approximation, we can obtain mathematical solutions of the model's degree distribution, degree-dependent clustering coefficient, and average clustering coefficient, which were observed to depend on temperature in Reference [18]. The details are described in the Method section.

### Comparison between the model and real networks

Here, we compare structural properties between the proposed model and the real metabolic networks of 113 organisms (used in [18]), where ubiquitous metabolites such as water, NH_3_, and ATP are excluded from use in analysis. These metabolic networks are represented by undirected graphs in which nodes and edges correspond to metabolites and substrate-product relationships, respectively (see Method for details). We first obtained the parameters *p *and *q *from the metabolic network of each organism using Equations (14) and (17), respectively. Substituting the parameters into the mathematical solutions [Equations (4), (10), and (11)], which are shown in Method, we next obtain structural properties from this model.

**Figure 2:**
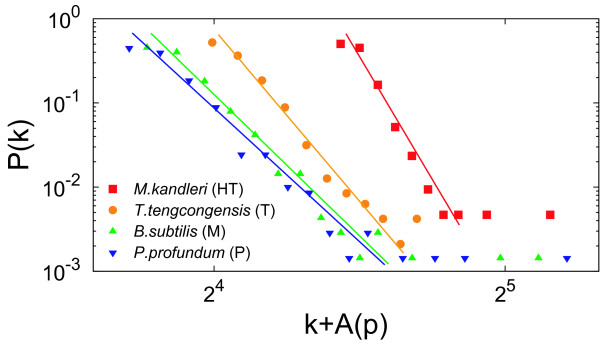
**Comparison of the degree distribution between our model and real metabolic networks**. The symbols indicate the real data. The lines are theoretical predictions from Equation (4).

Figure 2 shows a comparison of the degree distribution between our model and real metabolic networks. We provide *P *(*k*) for four organisms, which are selected from each temperature class. We let the horizontal axis be *k *+ *A*(*p*) to enhance clarity.

**Figure 3:**
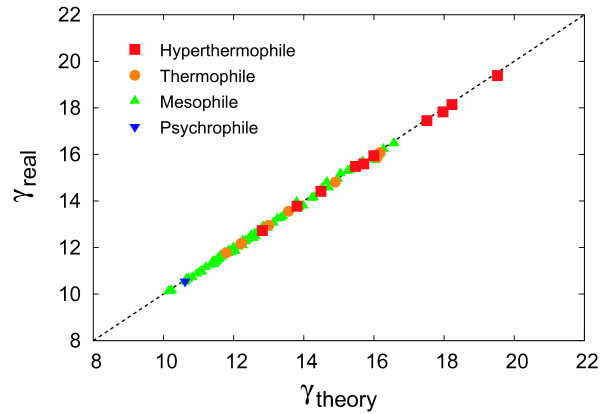
**Comparison of the degree exponent *γ *between our model (theory) and real metabolic networks (real)**. The dashed line shows *γ*_*real *_= *γ*_*theory*_.

Figure 3 shows a comparison of the degree exponent between our model and real metabolic networks. For comparison, the degree exponents are obtained by the maximum likelihood method considering a cutoff, which is the term *A*(*p*) (see Method for details). Note that this is different from the original maximum likelihood method [27] which does not include the coefficient *A*(*p*). For this reason, the observed values in Figure 3 are higher than that in Reference [18].

**Figure 4:**
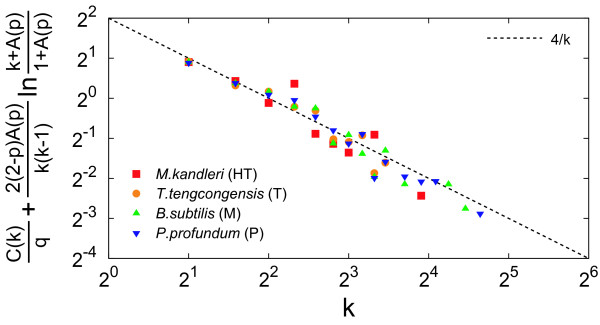
**Comparison of the degree-dependent clustering coefficient between our model (theory) and real metabolic networks (real)**. The symbols indicate the real data. The dashed line represents the theoretical prediction 4/*k*.

Figure 4 shows a comparison of the degree-dependent clustering coefficient between our model and real metabolic networks. We show *C*(*k*) for the same four organisms used in Figure 2. For clarity, we let the vertical axis be $\frac{C(k)}{q}+\frac{2(2-p)A(p)}{k(k-1)}\ln\frac{k+A(p)}{1+A(p)}$. If our model is reasonable, then the degree-dependent clustering coefficient of real networks follows 4/*k *[see Equation (10)].

**Figure 5:**
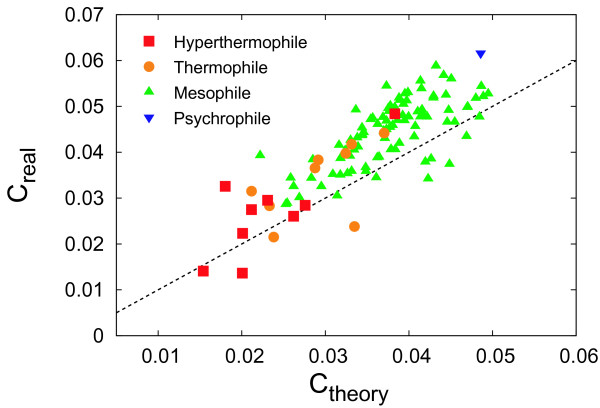
**Comparison of the average clustering coefficient *C *between our model (theory) and real metabolic networks (real)**. The dashed line shows *C*_*real *_= *C*_*theory*_.

Figure 5 shows a comparison of the average clustering coefficient between our model and real metabolic networks. The predicted average clustering coefficients are obtained with Equation (11) via numerical integral.

In addition, we also investigated clustering coefficients of a null model [28,29] (see also Method in details) in order to validate our model. Using this null model, we can obtain a null hypothesis for the clustering coefficients.

**Figure 6:**
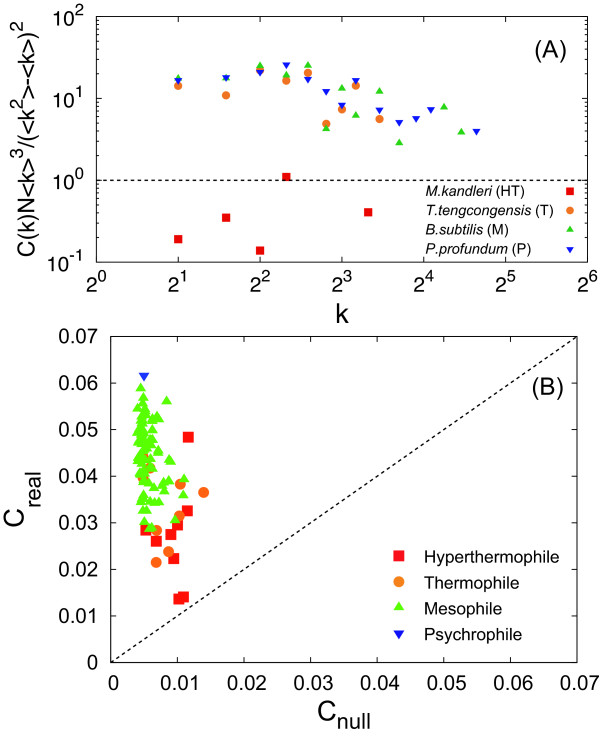
**Comparison of clustering coefficient between the null model and real metabolic networks**. (A) Degree-dependent clustering coefficient. The symbols indicate the real data. The dashed line represents the null hypothesis. (B) Average clustering coefficient. The dashed line shows *C*_*real *_= *C*_*null*_.

Figure 6(A) shows a comparison of the degree-dependent clustering coefficient between the null model and real metabolic networks. We show *C*(*k*) for the same four organisms. For clarity, the real *C*(*k*) is divided by *C*(*k*) of the null model; thus *C*(*k*)*N*⟨*k*⟩^3^/(⟨*k*^2^⟩ - ⟨*k*⟩)^2 ^[see Equation (19)]. If the null model reproduces real data, then the treated degree-dependent clustering coefficient should be 1.

Figure 6(B) shows a comparison of the average clustering coefficient between the null model and real metabolic networks. The average clustering coefficients of the null model are obtained with Equation (19). As shown in Figures 2, 3, 4, 5, the theoretical predictions are in good agreement with real data both qualitatively and quantitatively, indicating that this model can reproduce structural properties of real metabolic networks. As shown in Figure 6, in addition, the null model is significantly different from real data, further validating the reliability of our model.

### Relationship between model parameters and structural measures

In this section, we investigate a correlation between model parameters (*p *and *q*) and structural measures of metabolic networks in order to reveal the relationship between them.

Table 1 shows correlation coefficients between model parameters and structural measures.

**Table 1:** Correlation coefficient between model parameters and structural measures

	*p*	*q*	*γ*	*C*
Parameter *p*	-	-	-	-
Parameter *q*	0.30 (0.26)	-	-	-
Degree exponent *γ*	-0.93* (-0.88*)	-0.25 (-0.19)	-	-
Clustering coefficient *C*	0.68* (0.65*)	0.65* (0.58*)	-0.66* (-0.61*)	-

Each value denotes a Pearson's correlation coefficient. An adjacent value in parenthesis indicates a Spearman's rank correlation coefficient. The mark * by a correlation coefficient means that the *P *-value for the correlation is less than 0.001. Note that the degree exponent *γ *is obtained from Reference [18].

As shown in this table, there is a weak correlation between the parameters *p *and *q*. The parameters *p *and *q *control the emergence of short-cut paths and the length of a bypassed path, respectively. That is, this weak correlation implies that these mechanisms are virtually independent, suggesting the necessity of both mechanisms in the model.

The degree exponent *γ *has a strong negative correlation with the parameter *p *and a very weak correlation with the parameter *q*, implying that the degree exponent is dominantly influenced by the parameter *p*. This result is consistent with our analytical model [see Equation (5)]. On the other hand, the clustering coefficient correlates with both parameters *p *and *q*, being in agreement with our model [see Equations (10) and (11)].

In addition, we can observe a correlation between the degree exponent and the clustering coefficient. This correlation is due to the parameter *p *which indicates the mechanism: the emergence of short-cut paths. The degree exponent and the clustering coefficient reflect heterogenous connectivity and modularity, respectively. That is, this result suggests that these different structural properties, which are notably observed in metabolic networks, emerge via the same mechanism.

### Hypothesis from our model

In the previous two sections, we have shown that our model could reproduce real metabolic networks from diversified viewpoints. Therefore, we believe our model to be reliable, and we expect that we can discuss the origin of the structural difference in metabolic networks with respect to temperature via a correlation between the model parameters and optimal growth temperature.

**Figure 7:**
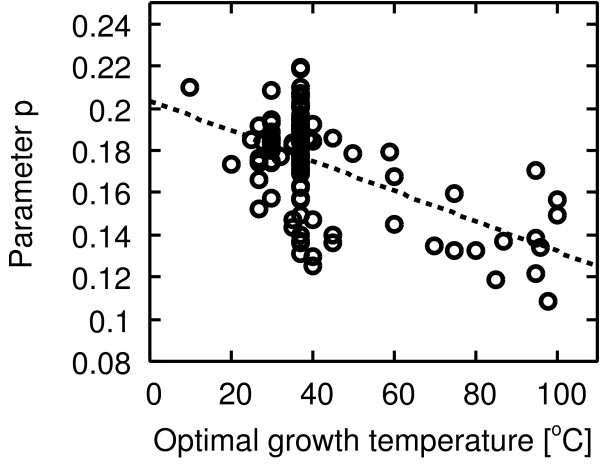
**Correlation between the parameter *p *and temperature**. Pearson's correlation *r *= -0.55 with *P *< 10^-9^, Spearman's rank correlation *r*_*s *_= -0.33 with *P *< 0.001.

**Figure 8:**
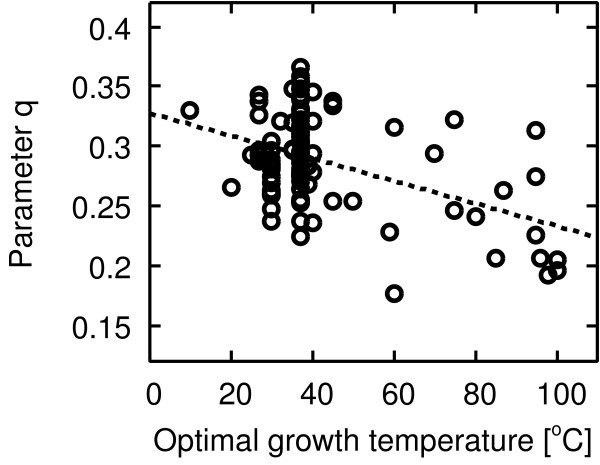
**Correlation between the parameter *q *and temperature**. Pearson's correlation *r *= -0.44 with *P *< 10^-5^, Spearman's rank correlation *r*_*s *_= -0.20 with *P *< 0.05.

Figures 7 and 8 show the negative correlation between temperature and the respective parameters *p *and *q *[see Additional file 1 for the parameters of each organism]. From this result, we speculate on the origin of structural difference with respect to temperature as follows.

In our model, the parameter *p *means the appearance frequency of the short-cut path between existing nodes. That is, the decay of the parameter *p *with temperature indicates that the emergence of the short-cut paths is inhibited at high temperature. This might be caused by strong selective constraints (negative selection) at high temperature [19,20].

The parameter *q *describes the length of bypassed path. A small value of *q *indicates that the bypassed path length is long. Therefore, the negative correlation between the parameter *q *and temperature implies that the short-cut path bypasses a relatively long path at high temperature.

### Cyclic properties in metabolic networks with respect to temperature

In the previous section, we obtained the following hypotheses from our model (based on the parameters *p *and *q*).

(i) The emergence of short-cut paths tends to be inhibited at high temperature.

(ii) However, when such a short-cut path does in fact emerge, the short-cut path is a bypass of a relatively long path at high temperature.

In order to show more convincing evidence of these hypotheses and therefore higher reliability of the model, here we investigate a relationship between cyclic properties of the metabolic networks and temperature. Since a cycle is generated due to the emergence of short-cut paths in our model, we can construe this hypothesis as

(1) The frequency of cycles is low at high temperature.

(2) The length of the cycle is relatively long at high temperature.

If our model is reliable, then we can observe these structural (cyclic) properties in real metabolic networks.

In order to investigate cyclic properties, we used the following metrics inspired by Reference [30]: the cycle index ⟨*r*^*c*^⟩ and the cycle length index ⟨*r*^*l*^⟩ (see Method for details). A high cycle index ⟨*r*^*c*^⟩ indicates a high frequency of cycles in a network. A high cycle length index ⟨*r*^*l*^⟩ means that the length of cycles tends to be short in a network (note that this does not depend on the frequency of cycles).

**Figure 9:**
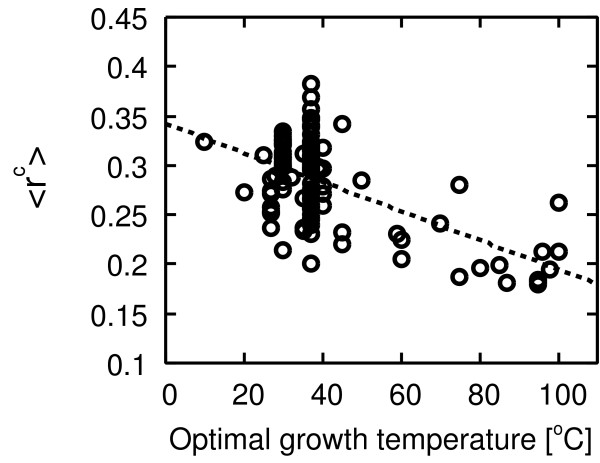
**Correlation between the cycle index and temperature**. Pearson's correlation *r *= -0.60 with *P *< 10^-11^, Spearman's rank correlation *r*_*s *_= -0.34 with *P *< 0.001.

**Figure 10:**
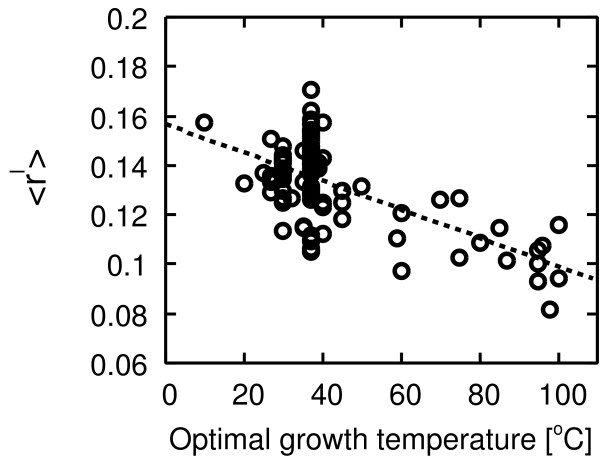
**Correlation between the cycle length index and temperature**. Pearson's correlation *r *= -0.63 with *P *< 10^-13^, Spearman's rank correlation *r*_*s *_= -0.33 with *P *< 0.001.

Figures 9 and 10 show the negative correlation between temperature and the respective indices ⟨ *r*^*c*^⟩ and ⟨*r*^*l*^⟩ [see Additional file 1 for the indices of each organism]. This result implies that the frequency of cycles becomes low with increasing temperature, and the length of the cycle increases with increasing temperature.

As above, the structural properties predicted from our model are also observed in real metabolic networks. This result implies more convincing evidence of our hypotheses and therefore higher reliability of the model.

## Discussion

We have proposed a simple model, which can reproduce the structural properties of real metabolic networks as shown in Figures 2, 3, 4, 5.

From this model, we have found that the structure of metabolic networks is determined by the emergence of short-cut paths. Our model contends that the emergence of the short-cut path is a possible origin of preferential attachment. Note that we do not directly use the preferential attachment. Although preferential attachment in metabolic networks has been revealed [31], its origin still not has been clarified. We believe that the short-cut mechanism we have demonstrated corresponds to the origin of the preferential attachment. In addition, the duplication and divergence model successfully explains the origin of the preferential attachment in protein interaction networks [32,33]. Moreover, the emergence of the short-cut path generates modules such as triangles and cycles whose length is more than 3. As shown in Figure 1, modules such as triangles and squares are merged into a network as a result. That is, this mechanism also corresponds to the merging module mechanism [34], which induces hierarchical modularity. In addition, these subgraphs might reflect network motifs [35,36] such as feedforward loops and bi-parallels because they correspond to triangles and squares in the case of undirected graphs. Thus, this mechanism is also a possible origin of the network motifs.

In this manner, the emergence of the short-cut path can explain the origin of several structural features: heterogeneous connectivity, network motifs (modules), and hierarchical modularity. We believe that this mechanism exists in real metabolic networks.

The correlations between the proposed parameters and temperature provide two hypotheses for structural difference with respect to temperature: the emergence of the short-cut paths is inhibited at high temperature, and the short-cut path is a bypass of a relatively long path at high temperature.

In order to show more convincing evidence of these hypotheses and the reliability of our model, we have also investigated cyclic properties of metabolic networks. If these hypotheses are correct, then we can observe the following cyclic properties in metabolic networks: the frequency of cycles is low at high temperature, and the length of a cycle is relatively long at high temperature. As shown in Figures 9 and 10, as expected, we have confirmed such cyclic properties. Therefore, our hypotheses are believed to be reliable. These cyclic properties are also novel temperature-dependent features in metabolic networks. Additionally, we can observe a variance among structural parameters in mesophiles. A possible reason of this variance is the effect of an organism's lifestyle. Temperature might be not the unique environmental factor in the network formation. Other factors might also influence the structure of metabolic networks. Parter *et al*. have reported that the modularity and other structural properties such as the clustering coefficient and cyclic coefficient [30] are different between different lifestyles [37]. When we consider one factor (temperature) only, we might see the variance because several factors influence the formation of metabolic networks.

We speculate on possible reasons of the two formation mechanisms, which are predicted from the model, in metabolic networks. First, we discuss why the emergence of the short-cut path is inhibited at high temperature (the correlation between the parameter *p *and temperature in Figure 8). This might be caused by a temperature-dependent selective constraint (negative selection) [19,20]. Enzymes (reactions) might need structural stability to survive in hot environments because enzymes tend to easily deactivate in such conditions. Metabolic networks are believed to evolve via evolutionary events such as gene duplication [21-23] and horizontal gene transfer [24]. Such evolutionary events consequently generate new enzymes. In the case of gene duplication, since the one of duplicated genes has to perform for the biological subsistence of the organism, the selective pressure against the other gene becomes weak [25]. As a result, the other gene, which codes for a new enzyme, tends to mutate due to weak selective pressure. Hence, due to gene duplication, the new enzyme might not successfully adapt to high temperature because the structural stability of the enzyme potentially becomes low due to mutations. On the other hand, new enzymes due to horizontal gene transfer might have no adaptation to high temperature because such genes, by which the new enzymes are coded, come from a different organism. In this manner, new reactions are hardly selected when new enzymes emerge via such evolutionary events because such enzymes might have no adaptation to high temperature. Therefore, we expect that the short-cut path tends to disappear because of the strong selective constraint at high temperature.

Next, we speculate why the short-cut path bypasses a relatively long path at high temperature (the correlation between the parameter *q *and temperature in Figure 8). This might be because there are less functionally similar enzymes at high temperatures. At high temperature, in our model, most of the new reactions are drawn between a new metabolite and an existing metabolite, indicating that the new enzyme tends to be functionally dissimilar to other enzymes. That is, the functionally dissimilar reactions (enzymes) lie in adjacent positions on a pathway. Therefore, in some cases, distances between functionally similar enzymes are long in a metabolic pathway. As a result, the short-cut path might bypass a relatively long path at high temperature when this path emerges. Of course, this is speculation, and in order to confirm this speculation, we need to more carefully test this hypothesis with a combination of biological sequence analysis and the Enzyme Commission (EC) number.

We finally summarize the origin of the structural difference in metabolic networks with respect to temperature. From our model, the emergence of the short-cut path is believed to determine structural properties such as the degree exponent and the clustering coefficient of metabolic networks. Therefore, the structural properties might change with temperature because this emergence is inhibited due to a temperature-dependent selective constraint.

We believe that the origin of structural difference with temperature provides new insights into the evolution of metabolic networks. Moreover, future studies in this line of research might contribute not only to a better understanding of evolutionary history but also to advancement of biotechnology such as detection and construction of organisms with temperature resistance, which have a great deal of potential in industry.

## Conclusion

We have proposed a simple model, which can reproduce the structural properties of real metabolic networks, in order to understand a possible origin of structural difference with respect to temperature in metabolic networks. We have found that the emergence of the short-cut path determines the structural properties. From our model, we have speculated that structural properties change with temperature because the emergence of the short-cut path tends to be inhibited due to strong selective constraint at high temperature. In addition, we have obtained a new hypothesis for design principles of metabolic networks: the short-cut path bypasses a relatively long path at high temperature if the new path emerges. We have shown additionally convincing evidence of these hypotheses and higher reliability of the model via network analysis.

## Methods

### Mathematical solution of the model

#### Degree distribution

First, we show an analytical solution for the degree distribution of the model via mean-field based analysis [38-40]. This analysis is based on a mean-field approximation, in which the many-body problem is considered as the one-body problem, and is widely used in the area of statistical mechanics of complex networks. Using the mean-field analysis, we can easily get the analytical solutions.

We here consider the time evolution of *k*_*i*_, which is the degree (the number of edges) of node *i*. The degree of node *i *increases by one with the probability 1/*N*, where *N *is the total number of nodes, when Event I (a new metabolite and new reaction) occurs. When Event II occurs, two existing nodes are selected, and their degrees increase respectively as follows. One node's degree increases by one with the probability 1/*N*, because this node is randomly selected. The other node's degree increases by one with the probability *k*_*i*_/∑_*j*_*k*_*j*_, because this node is selected by a random walk from the original randomly-selected node. It is reported that the probability that a walker arrives at this node equals *k*_*i*_/∑_*j*_*k*_*j *_irrespective of the number of steps in the random walk [41]. Note that this probability is equal to that of the probability in preferential attachment [38] which reproduces the heterogeneous connectivity. Thus, the time evolution of *k*_*i *_is

(1) $$\frac{\text{d}}{\text{d}t}k_{i}=(1-p)\frac{1}{N}+p\left[\frac{1}{N}+\frac{k_{i}}{\sum_{j}k_{j}}\right],$$

where *N *= (1 - *p*)*t *because the number of nodes increases by one with the probability 1 - *p*, and ∑_*j*_*k*_*j *_= 2*t *because one edge is added at every time. Note that this equation is independent of the bypassed path length (the parameter *q*). The solution of the above equation with the initial condition *k*_*i*_(*t *= *s*) = 1 is

(2) $$k_{i}=[A(p)+1]{\left(\frac{t}{s}\right)}^{p/2}-A(p),$$

where *A*(*p*) = 2/[*p*(1 - *p*)].

From the above equation, because *s/t *= *P *(≥ *k*), the cumulative distribution *P *(≥ *k*) is

(3) *P*(≥ *k*) = [*A*(*p*) + 1]^2/*p *^[*k *+ *A*(*p*)]^-2/*p*^.

Since $P(k)=-\frac{\text{d}}{\text{d}k}P(\geq k)$, finally, we get the degree distribution

(4) *P*(*k*) = (*γ *- 1) [*A*(*p*) + 1]^*γ *- 1 ^[*k *+ *A*(*p*)]^-*γ*^,

where the degree exponent *γ *is

(5) $$\gamma =\frac{2}{p}+1.$$

As shown in Equation (4), the degree distribution follows a power law with a cutoff within a small degree.

#### Degree-dependent clustering coefficient

Next, we show an analytical solution for the degree-dependent clustering coefficient of the model via mean-field analysis based on [39].

The clustering coefficient [10,12] of node *i *is defined as

(6) $$C_{i}=\frac{2M_{i}}{k_{i}\left(k_{i}-1\right)},$$

where *M*_*i *_is the number of edges among neighbors of node *i*. Here we consider the time evolution of *M*_*i*_. The number of edges *M*_*i *_increases with the probability *p *× *q*, because *M*_*i *_increases when Event II occurs and a path of length 2 is bypassed (a triangle is generated). That is, we do not need to consider a bypassed path of length greater than 2. Then, *M*_*i *_of each node, which belongs to the triangle, approximately increases by one. *M*_*i *_of one node increases by one with the probability 1/*N*, because this node is selected at random. *M*_*i*_s of the other two nodes increase by one with the probability *k*_*i*_/∑_*j*_*k*_*j*_, because these nodes are selected by a random walk. Therefore, the time evolution of *M*_*i *_is

(7) $$\frac{\text{d}}{\text{d}t}M_{i}≃pq\left[\frac{1}{N}+2\frac{k_{i}}{\sum_{j}k_{j}}\right],$$

where *N *= (1 - *p*)*t*, and ∑_*j*_*k*_*j *_= 2*t*. Moreover, *k*_*i *_= [*A*(*p*) + 1](*t/s*)^*p*/2 ^- *A*(*p*) as shown in Equation (2).

The solution of the above equation with the initial condition *M*_*i*_(*t *= *s*) = 0 is

(8) $$M_{i}=2q[A(p)+1]{\left(\frac{t}{s}\right)}^{p/2}+\frac{q(p-2)}{1-p}\ln\frac{t}{s}-2q[A(p)+1],$$

where *A*(*p*) = 2/[*p*(1 - *p*)]. From Equation (2), since *k*_*i *_= [*A*(*p*) + 1](*t/s*)^*p*/2 ^- *A*(*p*), this equation is rewritten as

(9) $$M_{i}=2q\left[\left(k_{i}-1\right)-\frac{1}{2}\left(2-p\right)A\left(p\right)\ln\frac{k_{i}+A\left(p\right)}{1+A\left(p\right)}\right].$$

Substituting this equation into Equation (6), we finally get the degree-dependent clustering coefficient

(10) $$C(k)=q\left[\frac{4}{k}-\frac{2\left(2-p\right)A\left(p\right)}{k\left(k-1\right)}\ln\frac{k+A\left(p\right)}{1+A\left(p\right)}\right].$$

#### Average clustering coefficient

Finally, we show a mathematical solution for the average clustering coefficient of the model. Since the average clustering coefficient is expressed as the summation of the product of the degree distribution and the degree-dependent clustering coefficient, it can be described as

(11) $$C=\int_{2}^{K_{m}}P\left(k\right)\times C\left(k\right)\text{d}k,$$

where *K*_*m *_is the maximum degree. The maximum degree is the case that the cumulative probability equals 1/*N *; thus *P*(≥ *K*_*m*_) = 1/*N*, and from Equation (3), *K*_*m *_can be expressed as

(12) *K*_*m *_= *N*^*p*/2^[*A*(*p *+ 1)] - *A*(*p*).

Equation (11) is solved via numerical integral because it is analytically unsolvable.

### Estimation of model parameters

This model has two parameters *p *and *q*. In order to reproduce structural properties in metabolic networks, we need to estimate these parameters in real-world networks. In this section, we show how to estimate the parameters.

#### The case of the parameter *p*

Here, we consider the average degree ⟨*k*⟩ of this model.

The average degree is defined as $\langle k\rangle =\frac{1}{N}\sum_{i}k_{i}$. As shown in the previous section, *N = *(1 - *p*)*t*, and ∑_*i*_*k*_*i *_= 2*t*. That is, the average degree of this model is

(13) $$\langle k\rangle =\frac{2t}{\left(1-p\right)t}=\frac{2}{1-p}.$$

From this equation, therefore, the parameter *p *is estimated by

(14) $$p=1-\frac{2}{\langle k\rangle },$$

where ⟨*k*⟩ is obtained from real metabolic networks.

#### The case of the parameter *q*

Here, we consider the number of triangles *T *of this model.

In this model, the number of triangles approximately increases by one with the probability *p *× *q *because a triangle is generated with the probability *q *when Event II occurs. That is,

(15) *T *≃ *pqt*.

Since *N *= (1 - *p*)*t*, this equation is rewritten as

(16) $$T≃pq\frac{N}{1-p}.$$

From this equation, therefore, the parameter *q *is estimated by

(17) $$q≃\frac{T}{N}\frac{1-p}{p},$$

where *T *and *N *are obtained from real metabolic networks.

### Data set

We used the metabolic networks of 113 organisms, which were previously investigated in Reference [18]. These metabolic networks are represented by undirected graphs in which nodes and edges correspond to metabolites and substrate-product relationships, respectively. For example, we consider a reaction S1+S2 → P1+P2. In this case, metabolites S1 and S2 connect to products P1 and P2, respectively. That is, the edge list is as follows: (S1, P1), (S1, P2), (S2, P1), (S2, P2). Note that if there are stoichiometric coefficients in the metabolic data used, then they are neglected. In order to accentuate constitutive pathways, these networks exclude 13 ubiquitous metabolites that serve for energy exchange, exchange of a proton or a phosphate moiety, and so on. To be exact, the following metabolites are excluded: water, ATP, ADP, NAD, NADH, NADPH, carbon dioxide, ammonia, sulfate, thioredoxin, (ortho) phosphate (P), pyrophosphate (PP), and H^+^. We only focused on the largest components of the metabolic networks in order to more accurately evaluate the structural properties.

### Maximum likelihood method considering a cutoff

In order to obtain the degree exponent from real metabolic networks, we used the maximum likelihood method [27]. However, this original method does not consider a cutoff, which we denote by the constant *A*(*p*), in the degree distribution. Thus, it is difficult to compare of the degree exponent between the model and the real data. Consequently, we consider an extended maximum likelihood method:

(18) $$\gamma =1+N{\left[\sum_{i=1}^{N}\ln\frac{k_{i}+A\left(p\right)}{k_{min}+A\left(p\right)}\right]}^{-1},$$

where *k*_*min *_is the minimum degree in a network.

### Null model

We used a null model to validate our model. The null model is an uncorrelated random scale-free network [28,29], and is a popular model. Assuming a power-law degree distribution, in the null model, we can obtain a null hypothesis for the degree-dependent clustering coefficient *C*(*k*) and the average clustering coefficient *C *using

(19) $$C\left(k\right)=C=\frac{{\left(\langle k^{2}\rangle -\langle k\rangle \right)}^{2}}{N{\langle k\rangle }^{3}},$$

where ⟨⋯⟩ denotes the average over all nodes. The values, ⟨*k*⟩, ⟨*k*^2^⟩, and *N*, are obtained from real metabolic networks.

### Indices for cyclic property

In order to characterize cyclic properties of networks, we define two indices inspired by the cyclic coefficient [30].

One is the cycle index of node *i*, defied as

(20) $$r_{i}^{c}=\frac{2}{k_{i}\left(k_{i}-1\right)}\sum_{\langle jh\rangle }R_{jh}^{i},$$

where

(21) $$R_{jh}^{i}=\{\begin{array}{ll} 1 & (\text{If there is at least one cycle}\\ & \text{that passes through node }i\text{ and its two neighbors }j\text{ and }h)\\ 0 & (\text{Otherwise}), \end{array}$$

and ⟨*jh*⟩ denotes all pairs of neighbors of node *i*. In addition, *k*_*i *_is the degree of node *i*. We can understand that this index is an extended clustering coefficient. This index considers cycles whose length is at least 3; however, the original clustering coefficient only focuses on cycles of length 3.

The second index is the cycle length index of node *i*, defined as

(22) $$r_{i}^{l}=\frac{\sum_{\langle jh\rangle }{\left(L_{jh}^{i}-2\right)}^{-1}}{\sum_{\langle jh\rangle }R_{jh}^{i}},$$

where $L_{jh}^{i}$ is the length of the smallest cycle that passes through node *i *and its two neighbors *j *and *h*.

In order to characterize global cyclic properties, in this section, we focus on the average indices $\langle r^{c}\rangle =\frac{1}{N}\sum_{i=1}^{N}r_{i}^{c}$ and $\langle r^{l}\rangle =\frac{1}{N}\sum_{i=1}^{N}r_{i}^{l}$, where *N *is the total number of nodes. Small values of ⟨*r*^*c*^⟩ indicate a low frequency of cycles in networks. Moreover, small ⟨*r*^*l*^⟩ means that the cycle length is globally long in networks.

### Ignoring cycles generated by the network representation

**Figure 11:**
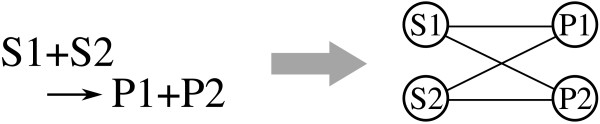
**An example of a cycle generated by the network representation**. When we consider a reaction S1+S2→P1+P2, we can see a cycle of length 4.

Using these indices as cyclic properties, we investigated the resulting cyclic properties in the metabolic networks of 113 organisms in order to test the hypotheses for cycles, which are generated due to the emergence of the short-cut path. However, we cannot directly use the metabolic networks, which are analyzed in Reference [18] because the metabolic networks include cycles, which are drawn by the network representation. For example, we consider a reaction S1+S2→P1+P2. In this case, a cycle of length 4, is generated as shown in Figure 11.

In this manner, cycles due to the network representation would be drawn when the types of all metabolites are different in a reaction and, as a result, the right-hand side and the left-hand side concurrently consist of multiple metabolites. Therefore, we ignored such cycles when calculated the cycle indices.

### Statistical analysis

In order to assess the significance of the observed correlations, we used Pearson's correlation coefficient *r*, Spearman's rank correlation coefficient *r*_*s*_, and their *P *-value *P*. We determine that there is a significant correlation between a structural property and optimal growth temperature when *P *< 0.05.

## Authors' contributions

KT conceived and designed the study. KT analyzed the mathematical solutions and the data, and drafted the manuscript. TA provided valuable discussions and suggestions during the development of the manuscript. KT and TA read and approved the final manuscript.

## Supplementary Material

Additional file 1 The estimated parameters and the cyclic indices of 113 organisms. This file includes the estimated parameters (*p *and *q*) and two cyclic indices of 113 organisms. In addition, the optimal growth temperature, the temperature class, and the domain for each organism also are included. The name of the organism is written according to KEGG (see [42] for full name). The "Domain" column indicates the type of domain of each organism (A: archea, B: bacterium). The "Temperature class" column represents the temperature class of each organism (HT: hyperthermophile, T: thermophile, M: mesophile, P: psychrophile). Click here for file

## References

[B1] Woese CR (1987). Bacterial evolution. Microbial Rev.

[B2] Pace NR (1991). Origin of life – Facing up to the physical setting. Cell.

[B3] Nisbet EG, Fowler CMR (1996). Some liked it hot. Nature.

[B4] Huang SL, Wu LC, Laing HK, Pan KT, Horng JT (2004). PGTdb: a database providing growth temperatures of prokaryotes. Bioinformatics.

[B5] Hickey DA, Singer GAC (2004). Genomic and proteomic adaptations to growth at high temperature. Genome Biol.

[B6] Kanehisa M, Araki M, Goto S, Hattori M, Hirakawa M, Itoh M, Katayama T, Kawashima S, Okuda S, Tokimatsu T (2008). KEGG for linking genomes to life and the environment. Nucleic Acids Res.

[B7] Albert R, Barabási A-L (2002). Statistical mechanics of complex networks. Rev Mod Phys.

[B8] Albert R (2005). Scale-free networks in cell biology. J Cell Sci.

[B9] Jeong H, Tombor B, Albert R, Oltvai ZN, Barabási A-L (2000). The large-scale organization of metabolic networks. Nature.

[B10] Watts DJ, Strogatz SH (1998). Collective dynamics of 'small-world' networks. Nature.

[B11] Wagner A, Fell DA (2001). The small world inside large metabolic networks. Proc R Soc Lond B.

[B12] Ravasz E, Somera AL, Mongru DA, Oltvai ZN, Barabási A-L (2000). Hierarchical organization of modularity in metabolic networks. Science.

[B13] Bollobas B (1985). Random Graphs.

[B14] Mendes JFF, Dorogovtsev SN (2003). Evolution of Networks: From Biological Nets to the Internet and WWW.

[B15] Barabási A-L, Oltvai ZN (2004). Network biology: Understanding the cell's functional organization. Nat Rev Genet.

[B16] Hartwell LH, Hopfield JJ, Leibler S, Murray AW (1999). From molecular to modular cell biology. Nature.

[B17] Ravasz E, Barabási A-L (2003). Hierarchical organization in complex networks. Phys Rev E.

[B18] Takemoto K, Nacher JC, Akutsu T (2007). Correlation between structure and temperature in prokaryotic metabolic networks. BMC Bioinformatics.

[B19] Wang H, Hickey DA (2002). Evidence for strong selective constraint acting on the nucleotide composition of 16S ribosomal RNA genes. Nucleic Acids Res.

[B20] Friedman R, Drake JW, Hughes AL (2004). Genome-wide patterns of nucleotide substitution reveal stringent functional constraints on the protein sequences of thermophiles. Genetics.

[B21] Horowitz NH (1945). On the evolution of biosynthesis. Proc Natl Acad Sci USA.

[B22] Papp B, Pál C, Hurst LD (2004). Metabolic network analysis of the causes and evolution of enzyme dispensability. Nature.

[B23] Díaz-Mejía JJ, Pérez-Rueda E, Segovia L (2007). A network perspective on the evolution of metabolism by gene duplication. Genome Biol.

[B24] Pál C, Papp B, Lercher MJ (2005). Adaptive evolution of bacterial metabolic networks by horizontal gene transfer. Nat Genet.

[B25] Ohno S (1970). Evolution by gene duplication.

[B26] Syvanen M (1985). Cross-species gene transfer; Implications for a new theory of evolution. J Theor Biol.

[B27] Newman MEJ (2005). Power laws, Pareto distributions and Zipf's law. Contemporary Phys.

[B28] Catanzaro M, Boguñá, Pastro-Satorras R (2005). Generating of uncorrelated random scale-free networks. Phys Rev E.

[B29] Newman MEJ, Strogatz SH, Watts DJ (2001). Random graphs with arbitrary degree distributions and their applications. Phys Rev E.

[B30] Kim H-J, Kim JM (2005). Cyclic topology in complex networks. Phys Rev E.

[B31] Light S, Kraulis P, Elofsson A (2005). Preferential attachment in the evolution of metabolic networks. BMC Genomics.

[B32] Vázquez A, Flammini A, Maritan A, Vespignani A (2002). Modeling of protein interaction networks. Complex Us.

[B33] Pastor-Satorras R, Smith E, Solé RV (2003). Evolving protein interaction networks through gene duplication. J Theor Biol.

[B34] Takemoto K, Oosawa C (2005). Evolving networks by merging cliques. Phys Rev E.

[B35] Alon U (2006). An Introduction to Systems Biology: Design Principles of Biological circuits.

[B36] Oosawa C, Takemoto K, Savageau MA (2008). Feedback and feedforward loops have opposite effects on dynamics of transcriptional regulatory model networks. Proceedings of the 13th International Symposium on Artificial Life and Robotics: 31 January – 2 February 2008; Beppu.

[B37] Parter M, Kashtan N, Alon U (2007). Environmental variability and modularity of bacterial metabolic networks. BMC Evol Biol.

[B38] Barabási A-L, Albert R, Jeong H (1999). Mean-field theory for scale-free random networks. Physica A.

[B39] Szabó G, Alava M, Kertész J (2003). Structural transitions in scale-free networks. Phys Rev E.

[B40] Barrat A, Pastor-Satorras R (2005). Rate equation approach for correlations in growing network models. Phys Rev E.

[B41] Saramäki J, Kaski K (2004). Scale-free networks generated by random walkers. Physica A.

[B42] KEGG organisms. http://www.genome.jp/kegg/catalog/org_list.html.

